# The Prognostic Importance of Changes in Renal Function during Treatment for Acute Heart Failure Depends on Admission Renal Function

**DOI:** 10.1371/journal.pone.0138579

**Published:** 2015-09-18

**Authors:** Ryan Reid, Justin A. Ezekowitz, Paul M. Brown, Finlay A. McAlister, Brian H. Rowe, Branko Braam

**Affiliations:** 1 Department of Medicine, Division of Nephrology, University of Alberta, Edmonton, Canada; 2 Division of Cardiology, University of Alberta, Edmonton, Canada; 3 Mazankowski Alberta Heart Institute, University of Alberta, Edmonton, Canada; 4 Canadian VIGOUR Centre, University of Alberta, Edmonton, Canada; 5 Patient Health Outcomes Research and Clinical Effectiveness Unit, University of Alberta, Edmonton, Canada; 6 Division of General Internal Medicine, University of Alberta, Edmonton, Canada; 7 Department of Emergency Medicine, University of Alberta, Edmonton, Canada; 8 School of Public Health, University of Alberta, Edmonton, Canada; 9 Department of Physiology, University of Alberta, Edmonton, Canada; University of Naples Federico II, ITALY

## Abstract

**Background:**

Worsening and improving renal function during acute heart failure have been associated with adverse outcomes but few studies have considered the admission level of renal function upon which these changes are superimposed.

**Objectives:**

The objective of this study was to evaluate definitions that incorporate both admission renal function and change in renal function.

**Methods:**

696 patients with acute heart failure with calculable eGFR were classified by admission renal function (Reduced [R, eGFR<45 ml/min] or Preserved [P, eGFR≥45 ml/min]) and change over hospital admission (worsening [WRF]: eGFR ≥20% decline; stable [SRF]; and improving [IRF]: eGFR ≥20% increase). The primary outcome was all-cause mortality. The prevalence of Pres and Red renal function was 47.8% and 52.2%. The frequency of R-WRF, R-SRF, and R-IRF was 11.4%, 28.7%, and 12.1%, respectively; the incidence of P-WRF, P-SRF, and P-IRF was 5.7%, 35.3%, and 6.8%, respectively. Survival was shorter for patients with R-WRF compared to R-IRF (median survival times 13.9 months (95%CI 7.7–24.9) and 32.5 months (95%CI 18.8–56.1), respectively), resulting in an acceleration factor of 2.3 (p = 0.016). Thus, an increase compared with a decrease in renal function was associated with greater than two times longer survival among patients with Reduced renal function.

## Introduction

In patients with chronic heart failure, co-existing chronic kidney disease is associated with poor outcomes [1–4] and vice versa.[5] In acute heart failure (AHF), more than 20% of patients are reported to experience worsening renal function (WRF)[1,6–8] and 11–18% to experience improved renal function (IRF) during hospitalization.[4,6,9] Although in-hospital WRF has been associated with worse outcome compared to patients with stable renal function (SRF).[6,7,10] Moreover, WRF poses a complex clinical problem from a nephrological perspective.[11] Fewer studies have investigated IRF and the association with outcomes.[6,9,12,13]

Importantly, renal function has been a focus for clinical trials enrolling patients with AHF, both as an efficacy and a safety endpoint.[14–16] Present definitions of WRF, SRF and IRF exclusively consider the magnitude of change in estimated glomerular filtration rate (eGFR; e.g., ≥20% decline), and do not incorporate admission renal function.[1,6,8,10,17] Definitions that do not incorporate a metric of ‘clinically relevant renal dysfunction’ may lead to overestimation of WRF and IRF incidence and underestimation of the prognostic importance of each. In addition, an eGFR <45 mL/min has been associated with significant consequences of renal dysfunction and elevated risk of hospitalization, cardiovascular events and all-cause mortality.[1,7,8,18–22]

The objective of this study was to evaluate the incidence of WRF and IRF using a definition that incorporates both admission renal function and change in renal function while in hospital. Furthermore, we evaluated the association between these definitions and long-term clinical outcomes in patients with AHF.

## Methods

### Study population

The Acute Heart Failure—Emergency Management (AHF-EM) study, conducted in Edmonton, Canada, prospectively enrolled patients with suspected AHF between June 2009 and November 2012 from four clinical sites: a stand-alone emergency department (ED), a community hospital, and two teaching hospitals. Patients were eligible if they were ≥18 years old, had suspected AHF and gave voluntary, informed written consent. Patients were excluded if they were already enrolled in a different AHF study, were on hemodialysis, or suffered from non-cardiac dyspnea, acute coronary syndrome, aortic dissection, or severe dementia. In total, 952 patients were enrolled in AHF-EM.

For the current analysis, participants who had an adjudicated diagnosis of AHF (see below) and for whom eGFR was calculable were included. The Chronic Kidney Disease Epidemiology Collaboration (CKD-EPI) equation (serum creatinine-based) was used to calculate eGFR at admission and discharge.[23]

### Adjudication

Two cardiologists who were unaware of patient outcomes independently adjudicated each patient’s diagnosis via detailed chart review (i.e., echocardiographic, radiographic, and laboratory test results, clinical notes from throughout admission, and discharge summaries). The physicians categorized each patient’s presenting diagnosis as AHF or not AHF. To assist in this adjudication, the Carlson criteria were also used, which assigns a numeric score representing the likelihood that an ED visit is attributable to AHF (low [score <5], intermediate [score 5–7], and high [score ≥8] likelihood of AHF).[24]

### AHF-EM data

Demographic information, past medical history, clinical status at presentation, medications taken prior to admission, and laboratory and radiographic test results were collected from the patients’ charts. Some laboratory values were not systematically available; for example, B-type Natriuretic Peptide (BNP) is not routinely tested and was available for a minority of patients. Outcomes including all-cause mortality and subsequent hospital admission were recorded during the follow-up period.

Re-hospitalization, repeat ED visits and mortality were collected directly from the clinical chart, and supplemented by administrative data from Alberta Health Services—Data Integration Measurement and Reporting using the International Classification of Disease (ICD) 10^th^ coding.[12] Administrative data included primary (first coding field) and secondary ICD-10 diagnoses of heart failure (I50.x), and were derived from the Ambulatory Care Classification System, which identifies one primary and up to 9 other diagnosis codes and 5 procedures for patients who visit an ED or hospital based outpatient clinic in the province of Alberta. Additionally, we used the Discharge Abstract Database, which is based on extractions from hospital discharge summaries and provides up to 25 diagnosis codes and 10 procedures per hospitalization.

### Renal function definitions

All definitions compare CKD-EPI eGFR at admission and discharge. Reduced (R) renal function was defined as an admission and/or discharge eGFR <45 mL/min/1.73 m^2^, and Preserved (P) renal function was defined as both admission and discharge eGFR ≥45 mL/min/1.73 m^2^. Using admission and discharge eGFR, WRF was defined as eGFR decline ≥20%, IRF was defined as eGFR increase ≥20%, and SRF was defined as less than 20% change. Percent-change calculations reference the admission value. These definitions were then combined to create 6 independent subgroups (complete definitions provided in S1 Table).

For example, R-WRF was defined as discharge eGFR <45 mL/min/1.73 m^2^ and eGFR decline ≥20%. The R-SRF group includes patients with <20% eGFR change and admission and/or discharge eGFR <45 mL/min/1.73 m^2^. The R-SRF also includes patients with eGFR <15 mL/min/1.73 m^2^ at both admission and discharge, regardless of proportional change in eGFR. In order to compare R-WRF/R-IRF to a conventional WRF/IRF definition, we also identified WRF and IRF groups with ≥20% eGFR change, with no requirement regarding admission renal function.[7]

### Statistical methods

Continuous variables were summarized using median (interquartile range [IQR]). For continuous variables the Kruskal-Wallis test was used to compare the six groups. Categorical baseline characteristics were summarized using frequency (%) and the subgroups were analyzed using the Pearson χ^2^ test.

**Fig 1:**
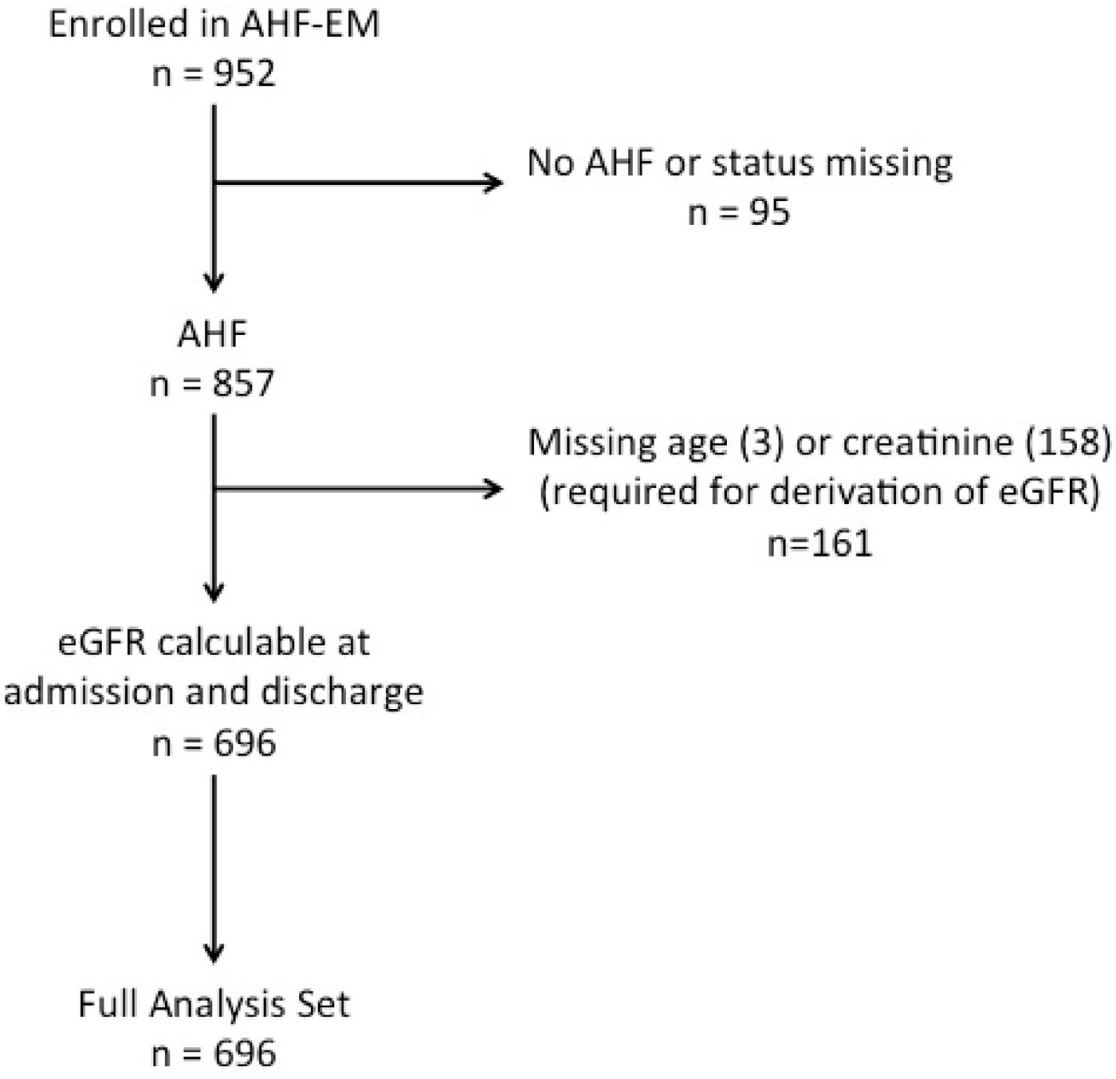
Patient accountability.

The outcome variable was time from admission to death (all-cause mortality). Up to five-year survival data were available; surviving patients had their survival time censored at the time of analysis. The LIFETEST procedure in SAS was used to obtain the Kaplan-Meier (KM) estimates of S(t), with the Wilcoxon test used for the comparison of survival curves. The proportional hazards assumption was violated (according to a formal statistical test and visual inspection of the log-cumulative hazard plot). An accelerated failure time (AFT) model [14] with an extended generalized γ (EGG) [18–22,25] distribution was estimated using the LIFEREG procedure (maximum likelihood method), thus enabling comparison of the renal function groups and the adjustment of covariates (the generalized γ was selected based on a plot of the empirical hazards and model fit i.e. -2logL). Cox-Snell residuals and a plot of the KM estimates against model estimates of S(t) indicated good agreement between model and empirical estimates (Fig 1). In an *a priori* analysis, covariates identified as clinically important (including variables from other published risk adjustment models and variables in Table 1) were retained if the corresponding p-value was <0.2, and amongst others, include age, sex, respiratory rate at ED, heart rate at ED, systolic blood pressure, serum sodium, prior chronic obstructive pulmonary disease (COPD), prior diabetes mellitus. BNP was not included in the multivariable model due to its missingness of 10.3% which was unequally distributed between renal function categories. AFT results are in terms of the ratio of percentiles or the Acceleration Factor (the ratio of percentiles); a ratio = 1 indicates no difference in survival, >1 indicates prolonged survival compared to the comparator group. Hypotheses of interest where: comparison of survival between Reduced and Preserved patients, and between IRF, WRF and SRF for patients with Reduced and Preserved renal function separately.

**Table 1:** Baseline characteristics.

	Total cohort	Reduced (52.2%)			Preserved (47.8%)			
		WRF	SRF	IRF	WRF	SRF	IRF	p-value
	N = 696	N = 79	N = 200	N = 84	N = 40	N = 246	N = 47	
	100%	11.4%	28.7%	12.1%	5.7%	35.3%	6.8%	
**Demographics**								
Age, years	77 (67, 84.5)	80.0 (73, 87)	80 (73, 85)	79 (68, 86)	72.5 (60, 81)	74 (61, 83)	71 (62, 78)	<0.0001
Male, n (%)	368 (52.9)	29 (36.7)	104 (52.0)	31 (36.9)	29 (72.5)	139 (56.5)	36 (76.6)	<0.0001
**Clinical Status at Presentation**								
Heart rate, beats/min	84.0 (70.0,103.5)	82.0 (70.0,102.0)	79.0 (66.0, 96.0)	82.0 (64.0,105.5)	88.0 (69.5,103.5)	88.5 (73.0,106.0)	94.0 (73.0,108.0)	0.0008
Systolic BP, mmHg	133.5 (118,150)	144 (122,153)	133 (120,149)	124 (110,144.5)	141 (126.5,158)	136 (117,153)	124 (112,146)	0.0005
Diastolic BP, mmHg	75.0 (64, 88)	72.0 (62, 88)	71.0 (62, 84)	68.5 (56.5, 82.5)	76.5 (67, 93.5)	78 (66, 90)	78 (69, 90)	<0.0001
Respiratory rate, breaths/min	22 (20, 26)	24 (20, 28)	22 (20, 26)	20 (18, 24)	24 (20, 30)	22 (20, 28)	22 (20, 25)	0.2333
Weight, Kg	81.5 (67.2, 97.3)	77.0 (57.0,100.0)	81.5 (64.0, 90.5)	82.0 (68.0, 95.0)	81.8 (75.0,104.2)	81.5 (68.0,100.9)	84.2 (70.0,105.0)	0.5362
Elevated JVP, n (%)	540 (90.6)	56 (88.9)	159 (94.1)	66 (90.4)	30 (96.8)	190 (87.6)	39 (90.7)	0.2713
Leg/Sacral edema, n (%)	531 (79.4)	66 (85.7)	152 (78.4)	71 (87.7)	28 (70.0)	187 (80.3)	27 (61.4)	0.0058
**Heart Failure and Cardiovascular History, n(%)**								
Ischemic etiology	278 (39.9)	33 (41.8)	99 (49.5)	36 (42.9)	13 (32.5)	80 (32.5)	17 (36.2)	0.0109
Valvular etiology	139 (20.0)	22 (27.8)	40 (20.0)	22 (26.2)	3 (7.5)	46 (18.7)	6 (12.8)	0.0562
Hypertensive etiology	323 (46.4)	43 (54.4)	105 (52.5)	37 (44.0)	21 (52.5)	99 (40.2)	18 (38.3)	0.0552
Arrhythmia (Atrial Fib/Flutter)	360 (51.7)	46 (58.2)	112 (56.0)	47 (56.0)	23 (57.5)	109 (44.3)	23 (48.9)	0.0907
Ejection fraction, %	45 (30, 55)	47.5 (31.5, 55)	45 (32, 55)	45.5 (35, 56)	40 (27, 52.5)	40 (27, 55)	40 (22.5, 52.5)	0.7282
**Medical History, n (%)**								
Diabetes mellitus	261 (37.7)	37 (47.4)	88 (44.0)	29 (34.9)	13 (32.5)	81 (33.1)	13 (28.3)	0.0467
COPD	227 (32.6)	27 (34.2)	68 (34.0)	29 (34.5)	13 (32.5)	74 (30.1)	16 (34.0)	0.9483
Hypertension	517 (74.9)	66 (84.6)	162 (81.4)	63 (75.9)	27 (67.5)	169 (69.3)	30 (65.2)	0.0067
**Medication, n (%)**								
ARB	101 (14.5)	11 (13.9)	36 (18.0)	8 (9.5)	5 (12.5)	37 (15.0)	4 (8.5)	0.3898
ACEi	393 (56.5)	36 (45.6)	88 (44.0)	43 (51.2)	28 (70.0)	164 (66.7)	34 (72.3)	<0.0001
Mineralocorticoid antagonist	111 (15.9)	7 (8.9)	22 (11.0)	14 (16.7)	8 (20.0)	44 (17.9)	16 (34.0)	0.0017
Beta-blocker	552 (79.3)	57 (72.2)	159 (79.5)	60 (71.4)	34 (85.0)	206 (83.7)	36 (76.6)	0.0876
Warfarin	277 (39.8)	27 (34.2)	79 (39.5)	35 (41.7)	15 (37.5)	102 (41.5)	19 (40.4)	0.9070
Aspirin	419 (60.2)	50 (63.3)	121 (60.5)	53 (63.1)	22 (55.0)	146 (59.3)	27 (57.4)	0.9359
Diuretics	612 (87.9)	67 (84.8)	170 (85.0)	74 (88.1)	36 (90.0)	221 (89.8)	44 (93.6)	0.4431
IV Loop Diuretic	589 (84.6)	65 (82.3)	177 (88.5)	67 (79.8)	33 (82.5)	209 (85.0)	38 (80.9)	0.4352
Digoxin	105 (15.1)	9 (11.4)	27 (13.5)	14 (16.7)	5 (12.5)	37 (15.0)	13 (27.7)	0.1921
CCB	163 (23.4)	26 (32.9)	53 (26.5)	17 (20.2)	5 (12.5)	54 (22.0)	8 (17.0)	0.0897
**Laboratory values**								
Hemoglobin, g/L	119 (104,134)	114 (102,128)	113 (102,126)	113 (97,127)	119.5 (109,136)	124 (111,140)	131 (113,147)	<0.0001
Sodium, mmol/L	138 (135,140)	138 (135,140)	138 (136,140)	137 (133,140)	136 (131.5,139)	138 (136,140)	139 (134,140)	0.0103
Potassium, mmol/L	4.1 (3.7, 4.5)	4.1 (3.7, 4.5)	4.2 (3.9, 4.7)	4.4 (3.9, 4.9)	4.1 (3.7, 4.3)	4.0 (3.7, 4.3)	4.1 (3.8, 4.5)	<0.0001
Creatinine, umol/L	110 (85,150)	115 (90,151)	149.5 (125.5,190)	164 (136.5,207.5)	81 (74.5, 92)	83 (69, 96)	108 (95,123)	<0.0001
BUN, mmol/L	8.7 (6.3, 13.4)	10.0 (7.8, 14.4)	12.1 (9.0, 17.6)	16.1 (11.4, 21.3)	5.4 (4.3, 7.1)	6.5 (5.0, 8.1)	8.3 (6.7, 10.0)	<0.0001
BNP, pg/ml*	1101 (638, 2021)	1474 (710, 2356)	1229 (753, 2223)	1217 (787, 2326)	942 (502, 1325)	908 (527, 1644)	1035 (826, 1800)	0.0004

For percentage calculations the denominator is the number of non-missing observations.

P-values are from Kruskal-Wallis or Pearson chi-square as appropriate.

Values are reported as median (Q1, Q3) or n (%) as appropriate.

ACEi, Angiotensin-converting-enzyme inhibitor; ARB, angiotensin II receptor blocker; BNP, b-type natriuretic peptide; BUN, blood urea nitrogen; COPD, chronic obstructive pulmonary disease; CCB, calcium channel blockers; IRF, improved renal function; JVP, jugular venous pressure; SRF, stable renal function; WRF, worsening renal function.

*BNP values available on 624 patients.

There was no imputation of missing data within the full analysis that included patients with AHF with a calculable eGFR at admission and discharge. No adjustment was made for multiple testing. A significance level of 0.05 was adopted as a guide for discerning differences between groups. Unless otherwise stated, SAS version 9.4 was used for all analyses.

### Ethics

The Health Research Ethics Board at the University of Alberta approved the study and all patients (or their proxies) provided informed written informed consent. The investigation conforms to the principles outlined in the Declaration of Helsinki.

## Results

### Cohort Characteristics

Of 952 enrolled patients, 857 (90%) patients had a confirmed diagnosis of AHF. Median follow-up time was 24.7 months (95%CI 23–27). 161 patients were excluded from further analysis because CKD-EPI eGFR on admission or discharge could not be calculated; these patients were similar in age, sex and other key clinical variables (data not shown). Therefore, the final analysis dataset consists of 696 patients (Fig 1). The characteristics of the study population are presented in Table 1. The median admission eGFR for the entire cohort was 49 mL/min/1.73m^2^.

**Fig 2:**
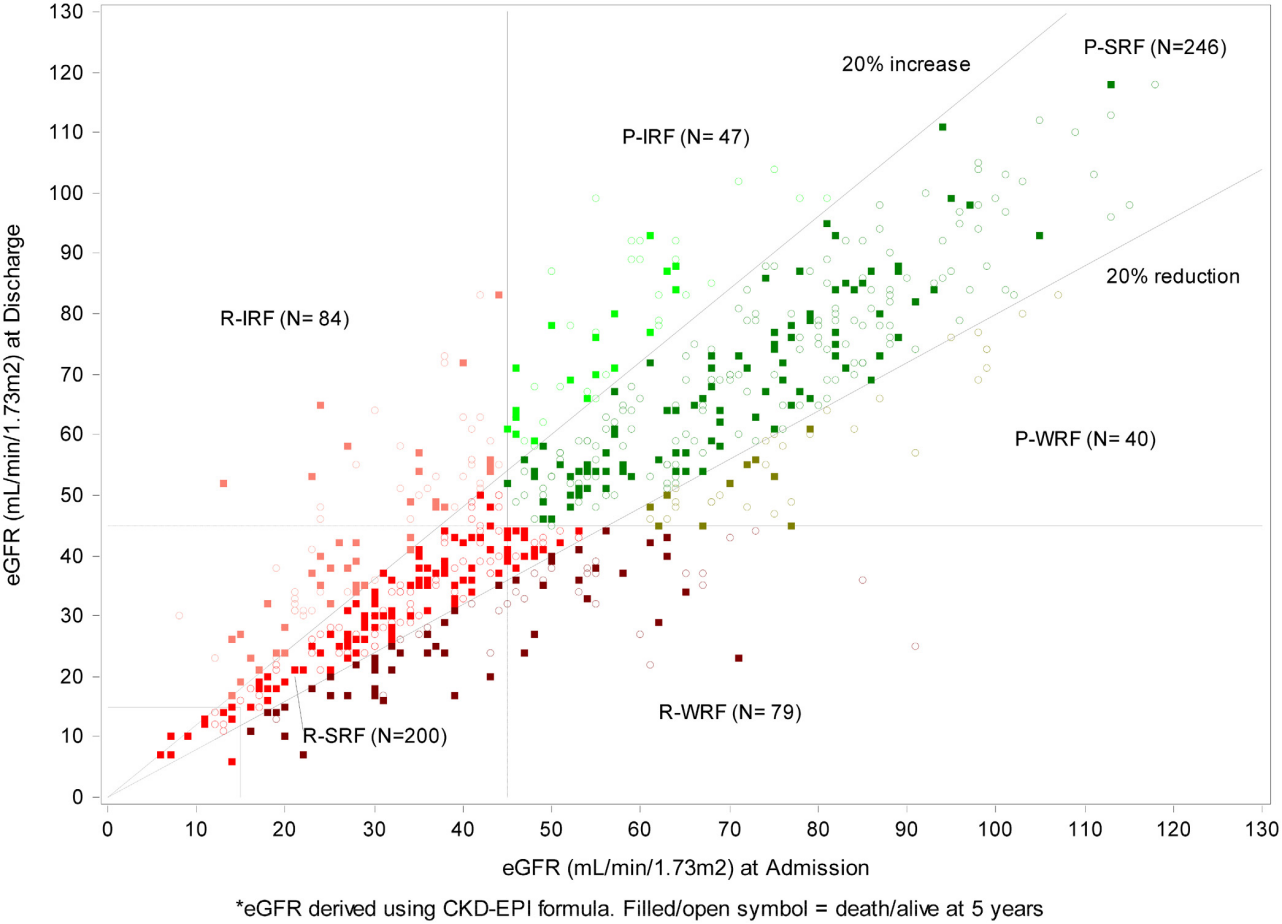
Scatter plot of admission versus discharge eGFR.

A scatterplot of admission versus discharge eGFR values is presented in Fig 2 and the distribution of the admission and discharge eGFR according to KDIGO (Kidney Disease Improving Global Outcome) staging criteria is presented in Table 2. The overall prevalence of preserved and reduced renal function was 47.8% and 52.2%, respectively. The overall incidence of worsening, stable or improved renal function for the whole cohort was 17.1%, 64.1% and 18.8%, respectively. The frequency of R-WRF, R-SRF, and R-IRF was 11.4%, 28.7%, and 12.1% respectively and the frequency of P-WRF, P-SRF, and P-IRF was 5.7%, 35.3%, and 6.8% respectively. Within the Reduced renal function group, the incidence of WRF, SRF and IRF was 21.7%, 55.1%, and 23.1%, respectively. Within the Preserved renal function group, the incidence of WRF, SRF and IRF was 12%, 73.8%, and 14.1%, respectively.

**Table 2:** KDIGO stages of patients at admission and discharge.

	Admission						
Discharge	G1	G2	G3a	G3b	G4	G5	*Total*
**G1**	25	14	2	0	0	0	41
**G2**	20	130	35	9	1	0	195
**G3a**	1	45	61	35	5	1	148
**G3b**	0	10	48	84	31	1	174
**G4**	1	4	2	36	72	4	119
**G5**	0	0	0	0	6	13	19
**Total**	47	203	148	164	115	19	696

G1: > = 90, G2: 60–89, G3a: 45–59, G3b: 30–44, G4: 15–29 ml/min/1.73m^2^, G5: <15 ml/min/1.73m^2^ or dialysis

Compared to the Preserved groups, the Reduced groups were older, were more often female, were less often on ACEi therapy and had lower hemoglobin levels (all p<0.05). Of note, factors associated with severity [e.g., blood pressures and signs of congestion (Jugular venous pressure (JVP), edema)] were similar between patients with Reduced and Preserved renal function.

### Differences between reduced and preserved renal function patients with IRF, SRF and WRF

Regardless of the level of renal function, groups with SRF had equal proportions of male and female patients; however, R-WRF and R-IRF patients were more likely to be female, while P-WRF and P-IRF patients were more likely to be male (Table 1). The distributions of comorbid conditions were similar across groups with the exceptions of diabetes, which was more common in patients with R-WRF and R-SRF.

There were small differences in vital signs and other parameters across renal function groups. Regardless of the level of renal function, patients with WRF had higher systolic blood pressure than patients in the IRF. Amongst those with Reduced renal function, blood urea nitrogen (BUN) was highest in the R-IRF (median 16.1, IQR: 11.4, 21.3) compared with the R-SRF (median 12.1, IQR: 9.0, 17.6) or the R-WRF (median 10.0, IQR: 7.8, 14.4). Patients in the R-IRF and P-IRF groups had similar BNP (medians 1217 vs. 1035 pg/ml). In contrast, BNP was higher in the R-WRF group than the P-WRF group (median 1474 vs. 942 pg/ml), and in the R-SRF group than the P-SRF group (median 1229 vs. 908 pg/ml).

### Outcome

**Fig 3:**
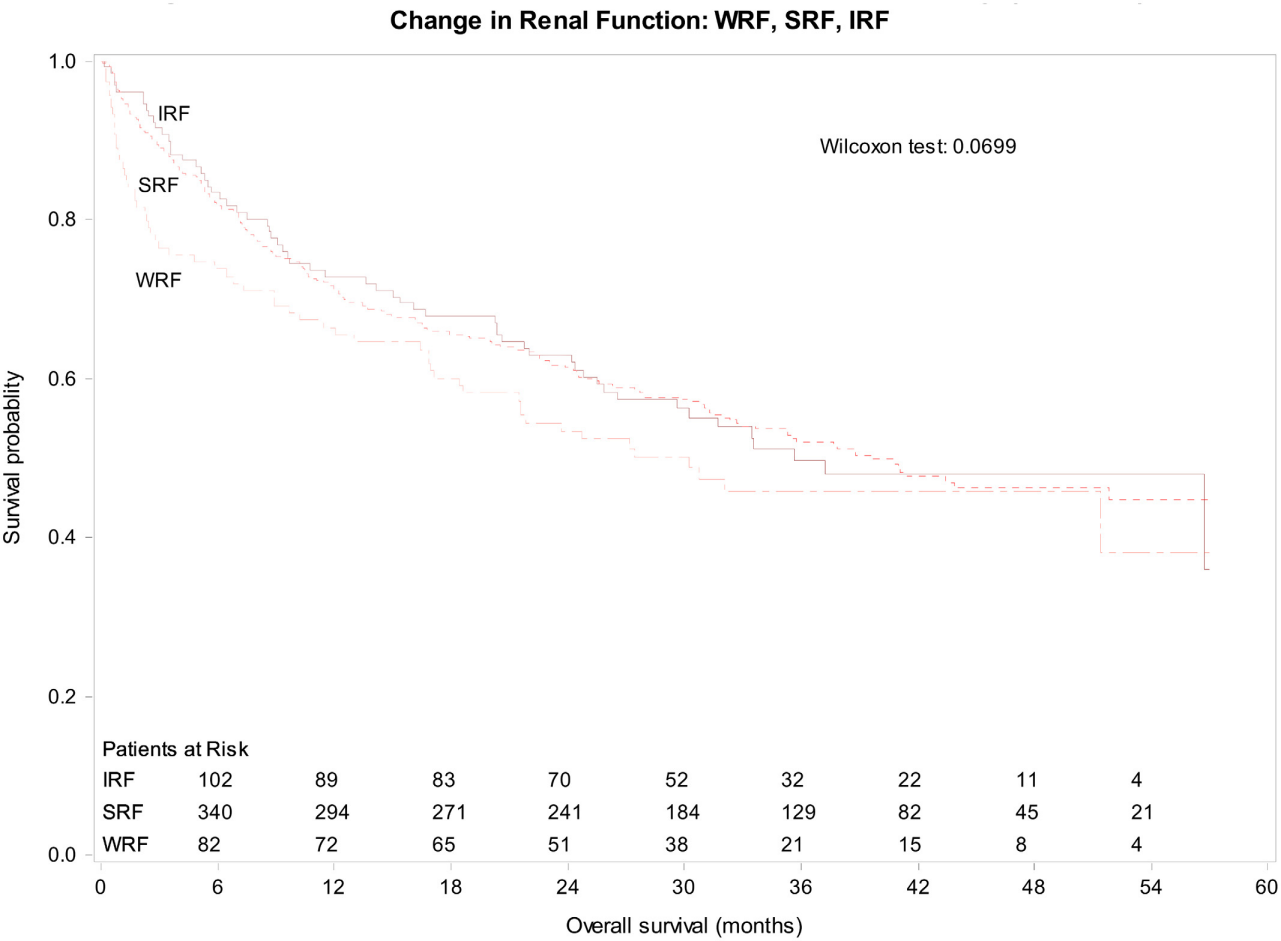
Kaplan-Meier Estimate for All-Cause Mortality over 5 years by changes in renal function. Legend: IRF = improved renal function; SRF = stable renal function; WRF = worsening renal function.

The death rate at 5 years was 47% with a median survival time of 37.7 months. The median survival times for Preserved and Reduced renal function were 68.4 months (95% CI 49–95) and 23.3 months (95% CI 17–31; p<0.0001), respectively. Median survival for IRF, SRF and WRF were 35.6, 39.6 and 30.2 months, respectively (Wilcoxon p-value = 0.07; Fig 3). The survival curves for the six renal function categories are shown in S1 Fig.

Survival of patients in the Reduced renal function groups was shorter for patients with WRF than IRF during admission, with median survival times of 13.9 months (95% CI 8–25) for R-WRF and 32.5 months (95% CI 19–56) for R-IRF, resulting in an acceleration factor of 2.3 (p = 0.016). Thus, patients with Reduced renal function have an association with longer survival by more than two times if they have an increase compared with a decrease in renal function during the index hospitalization. No difference in survival was seen between the P-WRF, P-SRF and P-IRF groups.

Using multivariable adjustment including clinically relevant covariates, and retaining in the model those covariates with corresponding p-value <0.2 (age, SBP, serum sodium and diabetes mellitus), the adjusted results were similar and statistically significant (Reduced vs. Preserved: p<0.0001; R-IRF vs. R-WRF p = 0.0034). Moreover, survival was worse for R-WRF when compared to R-SRF.

Table 3 presents the acceleration factors for different adjusted and unadjusted group comparisons (an acceleration factor >1 indicates extended survival for the group of interest). The acceleration factor for Preserved vs. Reduced is 2.9 (p<0.0001) indicating that survival for a patient with preserved renal function is approximately three times longer than that of a patient with reduced renal function.

**Table 3:** Results from Accelerated Failure Time Model* for All-Cause Mortality.

		Reduced (R)				Preserved (P)			
Statistic	Overall (N = 696)	IRF (N = 84)	SRF (N = 200)	WRF (N = 79)	Overall (N = 363)	IRF (N = 47)	SRF (N = 246)	WRF (N = 40)	Overall (N = 333)
No. of deaths (%)	325 (47)	42 (50)	112 (56)	49 (62)	203 (56)	18 (38)	93 (38)	11 (28)	122 (37)
Comparison†	P vs. R	I vs. S	S vs. W	I vs. W	I vs. S vs. W	I vs. S	S vs. W	I vs. W	I vs. S vs. W
Acceleration Factor, 95%CI	2.94, 2.05–4.20	1.38, 0.78–2.47	1.69, 0.95–3.02	2.34, 1.17–4.68		1.16, 0.52–2.59	0.95, 0.34–2.63	1.10, 0.32–3.81	
p-value	<0.0001	0.2712	0.0746	0.0161	0.0505	0.7092	0.9153	0.8789	0.9303
p-value**	<0.0001	0.1809	0.0280	0.0034	0.0117	0.9546	0.6601	0.7591	0.9075

^†^ Comparisons are first group vs. referent group. For example, for the P vs. R comparison, the Preserved group indicates that survival for a patient with preserved renal function is approximately three times longer than that of a patient with reduced renal function. For comparisons with 3 groups, no acceleration factor can be calculated.

*Parametric accelerated failure time model with a generalized gamma distribution.

**Adjusted for covariates showing p-value<0.2, namely: age, systolic BP, serum sodium, and prior diabetes mellitus.

## Discussion

Using data from a rigorous prospective study of 696 patients with AHF in the ED, and comparing WRF, SRF and IRF in the light of admission renal function, we identified two major findings. First, the incidence of both WRF and IRF is reduced considerably if only those patients with reduced renal function are considered. Secondly, the background renal function provided important context for interpreting any association of change in renal function with survival over a long-term follow-up period. For example, when compared to patients with IRF and SRF, WRF was only associated with shorter survival in patients with reduced renal function at admission. These findings have potential impact for the interpretation of completed and ongoing clinical trials as well as the development of clinical decision rules for admission to hospital.

In the literature, incidence of WRF in patients admitted for AHF is heavily dependent upon the criteria used to define WRF, which has not consistently included admission or discharge creatinine values. For example, Gottlieb and colleagues reported an incidence of WRF of 30% when applying a 20% increase in plasma creatinine; the incidence dropped to 11% if combined with an absolute decrease in renal function at discharge as defined by creatinine of >2.0 mg/dL.[23,26] Similarly, when the KDIGO Acute Kidney Injury Class I-III criteria (serum creatinine 1.5–1.9 times baseline or increase in serum creatinine ≥26.5 umol/L) were applied, 15% of patients with admission eGFR <60 ml/min/1.73m^2^ had AKI Class I-III.[24,27] Altogether, few studies used a relative change in eGFR together with a threshold decrease in admission eGFR in the WRF definition.[12,28] The incidence of WRF in 23 AHF studies was 27%,[28] considerably higher than the 11.4% of the patients in our study with R-WRF. Of the studies in a systematic review, the two studies using a >20% or >25% decrease in eGFR reported an incidence of 19% and 22%, respectively.[28]

Subgrouping the patient groups into Reduced and Preserved renal function led to the observation that WRF and IRF were not associated with adverse long-term outcome in patients with Preserved renal function. Conversely, in patients with Reduced renal function, there was a clear association of WRF with mortality as compared to SRF and IRF. In contrast to our study, Testani and coworkers report that controlling for admission eGFR caused IRF to lose its significant association with mortality.[4,7]

There is no consensus regarding the definition of clinically important acute changes in renal function in patients with AHF.[7,14,29,30] Given the absence of consensus on a definition for WRF, IRF or SRF, we are unable to directly compare risk in our study to others; our study suggests that admission renal function determines whether WRF is associated with poor outcomes. An implication of our study is that admission renal function needs to be included if WRF is to be considered as an endpoint for clinical trials.

Regarding IRF, there are no directly comparable studies to our incidence of 12.1% in the R-IRF group. Two studies used an increase in eGFR of >20% as a definition for IRF resulting in an incidence of 16% and 31% and in both cases linked this finding to poor outcomes.[4,6] However, both studies did not divide their groups based on absolute renal function (i.e. Preserved or Reduced renal function). In the first study, IRF during admission was associated with worse outcome more so if the IRF was still present at discharge.[4,27] In the second study, ‘dynamic’ renal function (IRF or WRF) was associated with worse outcome compared to SRF.[6,28] Our data are discordant with those observations. IRF was not associated with poorer outcome in the patients with Preserved renal function; rather, early survival was impaired, yet later survival seemed improved. In contrast, IRF in patients with Reduced renal function was associated with better survival compared to the WRF group.

Several key strengths and limitations of this study deserve attention. First, while we directly recruited patients in the ED with suspected AHF in order to increase the generalizability of our findings, the demographics of our study are similar to prior population-based studies.[4,12,13] Second, we had excluded 161 patients lacking paired creatinine values to calculate eGFR; however, these patients were similar to included patients. While only of modest size, our study had a 47% follow-up to 5-years and represents a generalizable, albeit higher risk, population. Third, we used an acceleration failure time model in order to account for the violation of the proportional hazards model. This may be of more than passing interest since absolute renal function and a change in renal function may have differential short or long-term effects on all-cause mortality and thus standard statistical models should account for this variation. We considered whether the proportional hazards assumption was valid within a shorter follow-up (e.g. 1 year), or after removing a particular group (e.g. P-WRF), but this was not the case, and thus the acceleration failure time model fit the data best including that of the presentation within and across groups.[7,14,29,30] The main results from the AFT model were also reiterated by the simple Wilcoxon test. Finally, the cutoff for reduced renal function was chosen because the prevalence of secondary issues of renal failure such as volume retention and hypertension increases steeply at the cutoff of 45 ml/min/1.73 m.[22]

This study underscores the relevance of admission renal function for interpreting acute changes in renal function during an AHF admission. WRF was associated with lower and IRF was associated with higher long-term survival in patients with reduced admission renal function; for patients with preserved admission renal function, acute changes in eGFR were not associated with poorer outcomes. Patients with R-WRF are a highly vulnerable group, and more precise characterization of the pathophysiology could lead to novel treatment options for this group.

## Supporting Information

S1 Fig Kaplan-Meier Estimate for All-Cause Mortality over 5 years by changes in renal function. Legend: IRF = improved renal function; SRF = stable renal function; WRF = worsening renal function.
(TIF)

S1 Table Definitions of renal function changes. (DOCX)
