# Supplementary figures and images for: The Prognostic Importance of Changes in Renal Function during Treatment for Acute Heart Failure Depends on Admission Renal Function
Source: PLoS One. 2015 Sep 18;10(9):e0138579. doi: 10.1371/journal.pone.0138579 (PMC4575105; doi:10.1371/journal.pone.0138579)

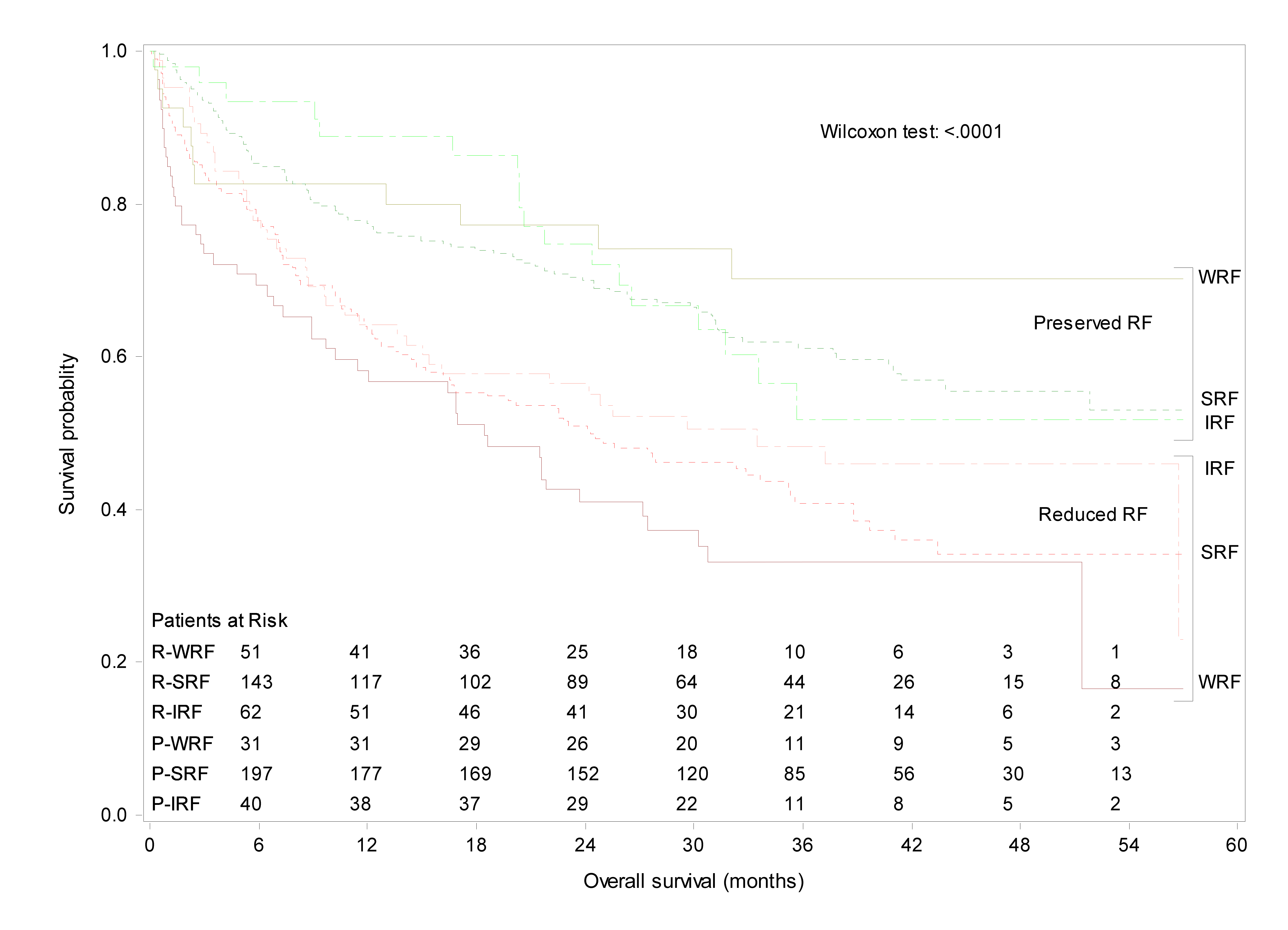

Supplement: S1 Fig — Legend: IRF = improved renal function; SRF = stable renal function; WRF = worsening renal function. (TIF) [file pone.0138579.s001.tif]
