# Supplementary material for: The Prognostic Importance of Changes in Renal Function during Treatment for Acute Heart Failure Depends on Admission Renal Function
Source: PLoS One. 2015 Sep 18;10(9):e0138579. doi: 10.1371/journal.pone.0138579 (PMC4575105; doi:10.1371/journal.pone.0138579)
Supplement: S1 Table — (DOCX) [file pone.0138579.s002.docx]

**Supplemental Table**

| Group | eGFR at admission / discharge |  | eGFR change |
| --- | --- | --- | --- |
| Preserved | admission and discharge eGFR ≥45 | IRF | increase ≥20% |
|  |  | WRF | decline ≥20%, |
|  |  | SRF | change < 20% |
| Reduced | admission and/or discharge eGFR <45 | IRF | increase ≥20% |
|  |  | WRF | decline ≥20%, |
|  |  | SRF | change < 20% |
